# Supplementary material for: Irreversibility in dynamical phases and transitions
Source: Nat Commun. 2021 Jan 15;12:392. doi: 10.1038/s41467-020-20281-2 (PMC7810704; doi:10.1038/s41467-020-20281-2)
Supplement: Supplementary file 1 — Supplementary Information [file 41467_2020_20281_MOESM1_ESM.pdf]

Irreversibility in dynamical phases and transitions

Supplementary Materials

SUPPLEMENTARY FIGURES

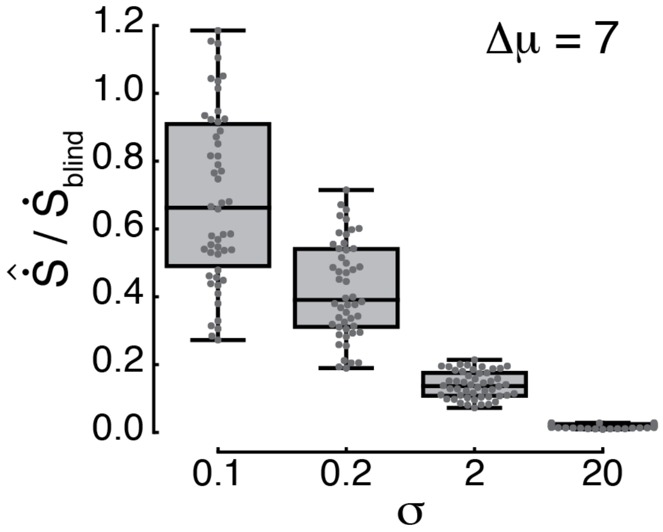

Supplementary Figure 1. **Effect of increasing smoothing width.** Underestimation of $\dot{S}_{\text{blind}}$ as a function of smoothing width, σ, for $\Delta\mu = 7$ for the Brusselator model introduced in the main text. We see a systematic decrease in the estimated EPR, $\hat{\dot{S}}$, as the smoothing width gets wider. Individual points show results for each of the $N = 50$ simulations. Center line shows the median, edges of the box show interquartile range, and whiskers show range of data.

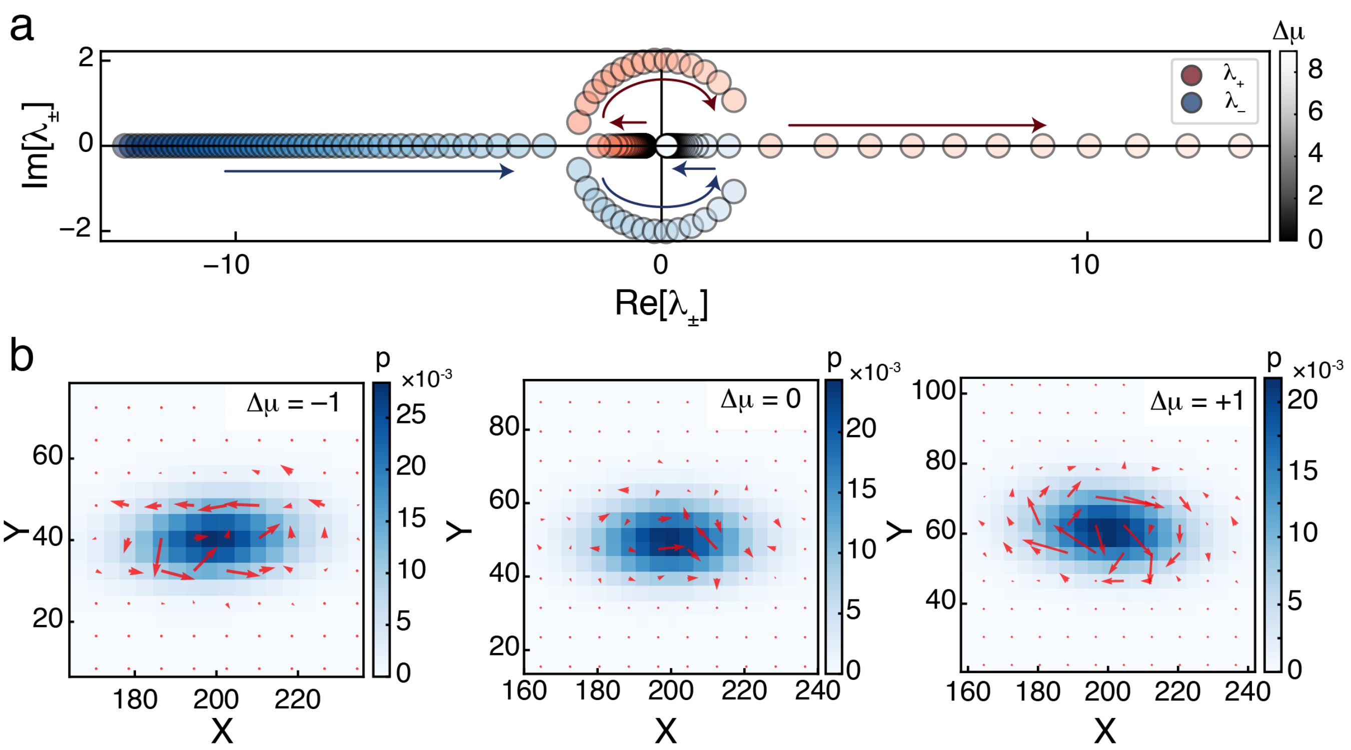

Supplementary Figure 2. **Brusselator dynamics exhibit circulation without macroscopic oscillatory solution.** (a) Eigenvalues of the Brusselator's relaxation matrix, R as a function of the chemical driving force, $\Delta\mu$. $\lambda_\pm$ shown in red and blue, respectively, with each color going from dark to light with increasing $\Delta\mu$. The red and blue arrows serve as guides for the reader to follow the trajectory of $\lambda_\pm$. With our parameters, the stable focus appears at $\Delta\mu = 5.26$ and the Hopf bifurcation occurs at $\Delta\mu_{\mathrm{HB}} = 6.16$. (b) Probability distributions (blue) and probability fluxes (red arrows) for Brusselator simulations with $\Delta\mu = [-1, 0, 1]$, showing the reversal in flux circulation direction at $\Delta\mu = 0$.

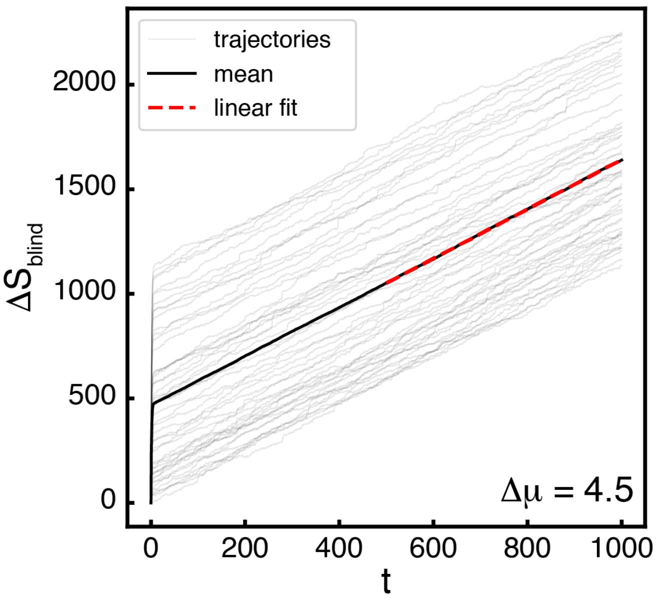

Supplementary Figure 3. **Calculating $\dot{S}_{\text{true}}$ and $\dot{S}_{\text{blind}}$** Fit to (blinded) entropy produced for Brusselator. Light gray lines show the amount of entropy produce as a function of simulation time for $N = 50$ simulations at $\Delta\mu = 4.5$. Each simulation starts at a random initial condition and rapidly approaches the steady state value for (X, Y). This transient trajectory results in the large variation in initial entropy production which depends on how far the system begins from $(X_{\text{ss}}, Y_{\text{ss}})$. Once the system reaches its steady state, the rate of entropy production approaches a steady value. The average of ΔS is taken across all trajectories, and a linear fit to the second half of the resulting mean gives us our value of $\dot{S}_{\text{blind}}$ given in Fig. 2b. The same method is used to calculate $\dot{S}_{\text{true}}$ as well as $\dot{s}_{\text{blind}}$ and $\dot{s}_{\text{true}}$ for the reaction-diffusion Brusselator model in Fig. 4.

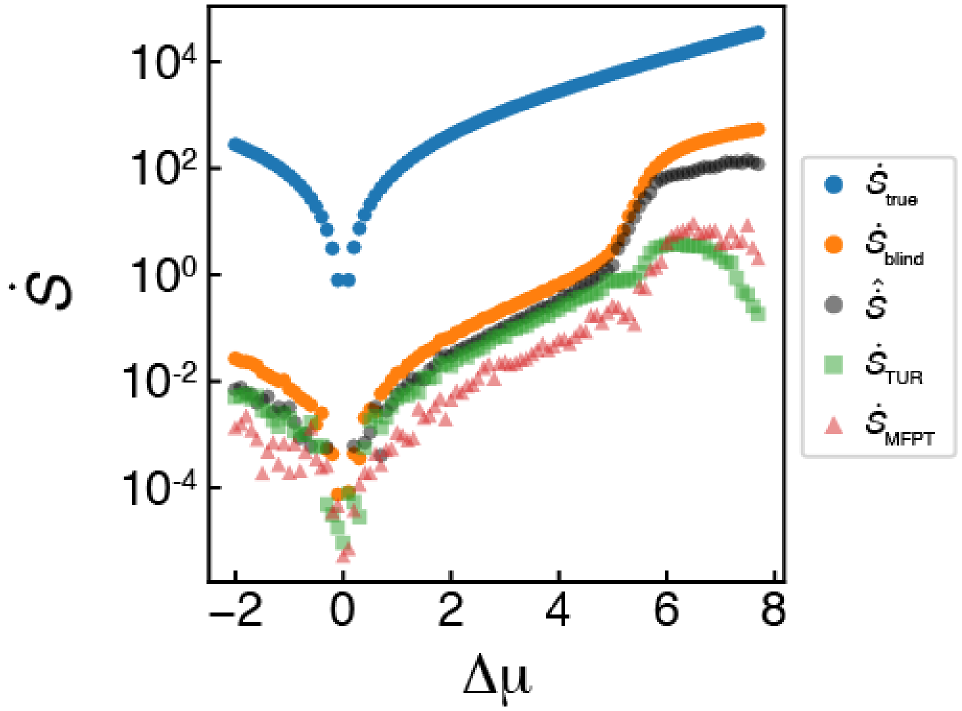

Supplementary Figure 4. **Alternative methods for measuring $\dot{S}$.** Comparison of $\hat{\dot{S}}$ (black dots, same data as in Fig. 2c) with two alternative methods for estimating entropy production rates. $\dot{S}_{\mathrm{TUR}}$ (green squares) is based on the thermodynamic uncertainty relation (TUR), and $\dot{S}_{\mathrm{MFPT}}$ is based on measuring the mean first passage time of an observable. All estimates approximate $\dot{S}_{\mathrm{blind}}$ because they are based only on observables in the (X, Y) plane. Our estimator, $\hat{\dot{S}}$, outperforms the other two estimators, especially beyond the Hopf bifurcation. See Supplementary Materials for details regarding the implementation of the TUR and MFPT methods.

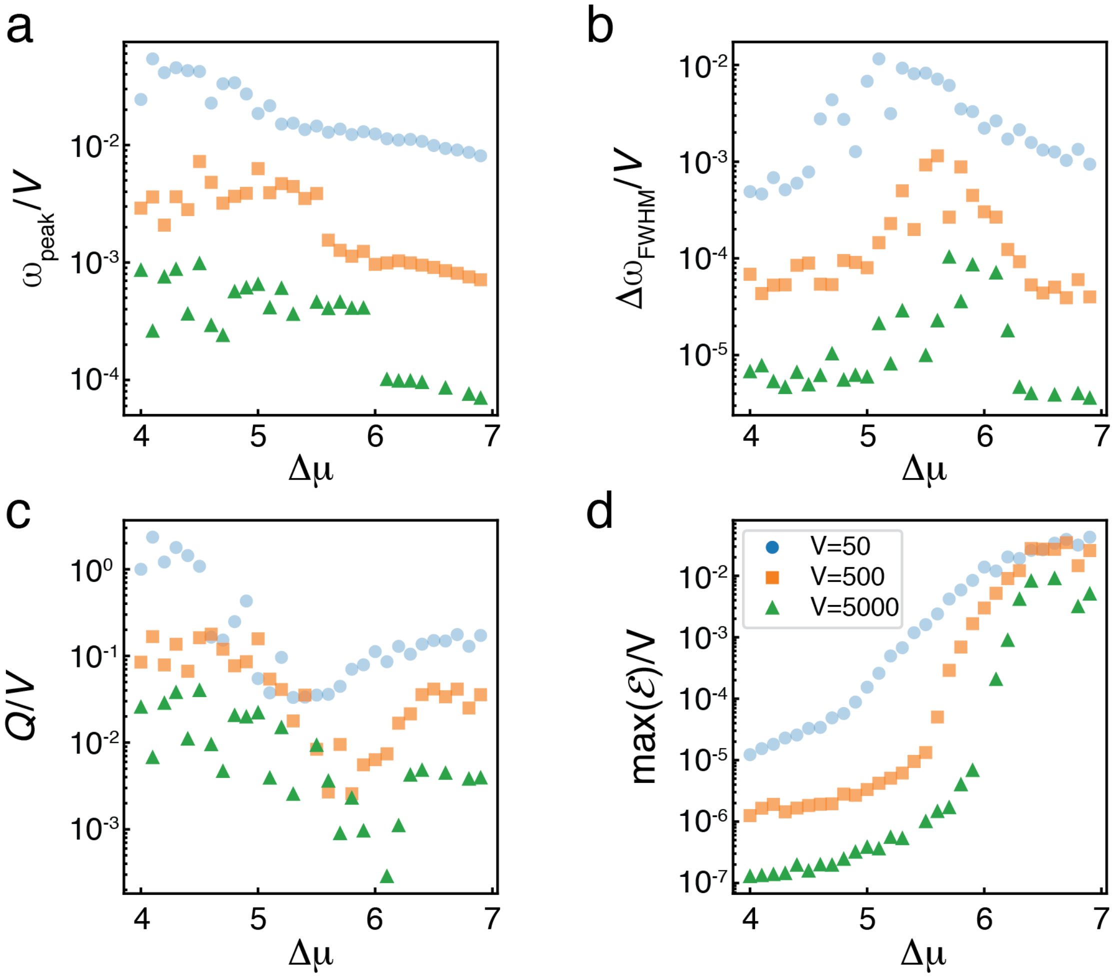

Supplementary Figure 5. **Finite size scaling of $\mathcal{E}$ of Brusselator.** (a) Normalized frequency of maximum of $\mathcal{E}$, ω_{peak}/V is independent of V, but the jump from high to low frequency occurs more sharply and occurs closer to $\Delta\mu_{\text{HB}}$ as V increases. (b) Normalized full-width half-maximum (FWHM) of peak in $\mathcal{E}$, $\Delta\omega_{\text{FWHM}}$, is independent of V and is maximized around the transition point, reflecting the increased fluctuations near the phase transition. The location of the peak moves closer to $\Delta\mu_{\text{HB}}$ as V increases. (c) The normalized quality factor of $\mathcal{E}$, $Q/V = \omega_{\text{peak}}/\Delta\omega_{\text{FWHM}}V$, is independent of system size, and has a minimum at the transition point. (d) The normalized maximum value of $\mathcal{E}$ is independent of V below the transition, and gains a linear dependence on V above it, similar to $\dot{S}_{\text{blind}}$.

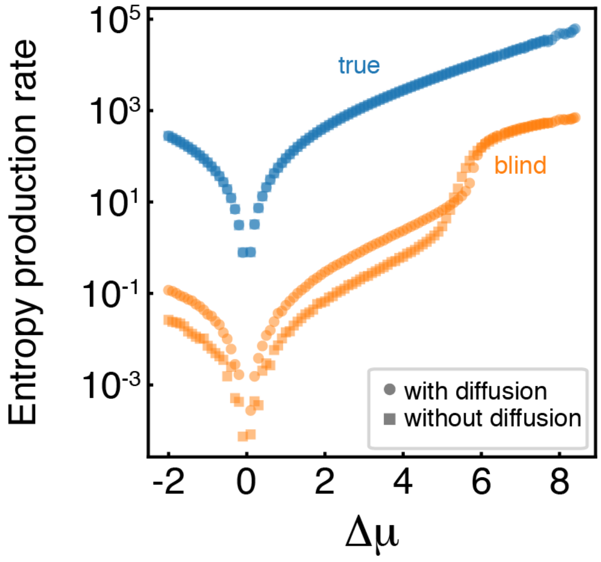

Supplementary Figure 6. **Comparing $\dot{S}$ and $\dot{s}$ for the well-mixed and reaction-diffusion Brusselator.** True (blue) and blind (orange) entropy production rates for the well-mixed Brusselator ($\dot{S}$, squares) and reaction-diffusion Brusselator ($\dot{s}$, circles). These are the same data shown in Fig. 2c and Fig. 4c, plotted together. $\dot{S}_{\text{true}} \approx \dot{s}_{\text{true}}$ for all driving forces $\Delta\mu$. By contrast, $\dot{s}_{\text{blind}} > \dot{S}_{\text{blind}}$ below $\Delta\mu_{\text{HB}}$ due to additional irreverisibility from diffusion between neighboring lattice sites with different concentrations of (X, Y) due to the incoherent dynamics, and $\dot{S}_{\text{blind}} \approx \dot{s}_{\text{blind}}$ above $\Delta\mu_{\text{HB}}$ due to the synchronized oscillations, making diffusion between lattice sites an equilibrium process.

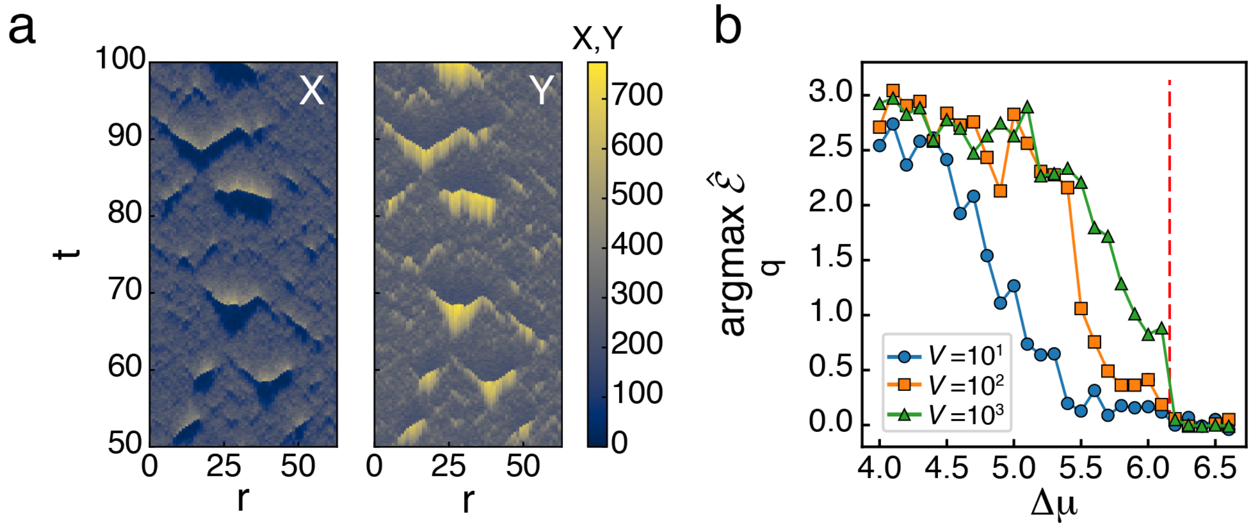

Supplementary Figure 7. **Transiently synchronized dynamics in the reaction-diffusion Brusselator and finite-size scaling in $\mathcal{E}$.** (a) Typical trajectory of a reaction-diffusion Brusselator system just below the Hopf bifurcation, at $\Delta\mu = 5.8$ and $V = 100$. See some flashes on collective behavior, but it does not span the entire system, showing why $\mathcal{E}$ has peaks somewhere between $q = 0$ and the maximum in Fig. 5c. (b) Wavenumber q that maximizes $\hat{\mathcal{E}}$ for the reaction-diffusion Brusselator for compartment volumes $V = \{10^1, 10^2, 10^3\}$ shows a sharper transition that gets closer to $\Delta\mu_{\text{HB}}$ (red line) as the volume increases.

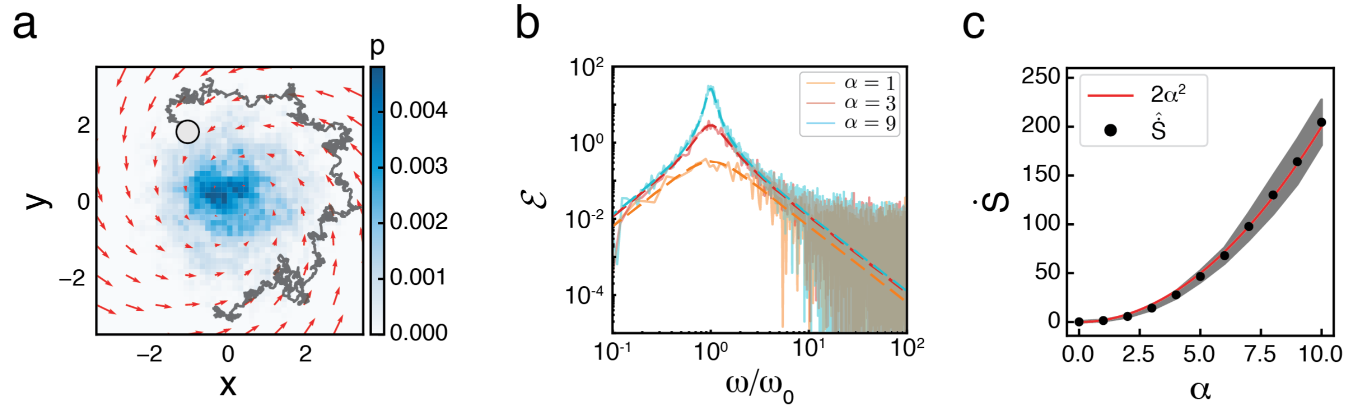

Supplementary Figure 8. $\dot{S}$ **of a driven Brownian particle.** (a) Sample trajectory for simulation of driven Brownian particle in 2 dimensions with $\alpha = 2$ in nondimensionalized units shown in gray with end of trajectory shown with white circle. The heatmap gives the empirical steady-state probability distribution function of particle positions and the red arrows indicate the underlying force field $F(\mathbf{x})$. (b) $\mathcal{E}$ for $\alpha = [1, 3, 9]$ measured from simulations and calculated from Equation 98, shown in solid and dashed lines respectively. $\mathcal{E}$ is symmetric in ω, so only the positive axis is shown. (c) EPR for $\alpha = \{0, 1, \ldots, 10\}$, smoothed by a Gaussian with $\sigma_\omega = 2.1$. Mean ± s.d. of $\hat{\dot{S}}$ over $N = 64$ simulations shown with black dots and shaded area. Red line shows non-dimensionalized theoretical value of $\dot{S}$. See Supplementary Materials for all simulation parameters.

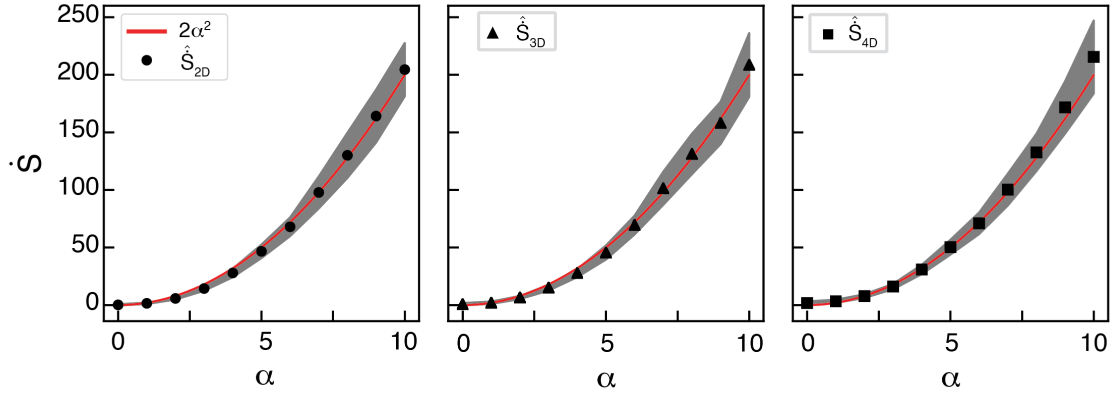

Supplementary Figure 9. $\dot{S}$ **of driven Brownian particle in higher dimensions.** $\hat{\dot{S}}$ for driven Brownian particle simulation in $d = [2, 3, 4]$. Additional dimensions only contain a harmonic potential term, maintaining the non-equilibrium driving only in the 2nd dimension. All other parameters the same as in Supplementary Figure 8.

SUPPLEMENTARY TABLES

name	value	units	description
N_{sim}	10	1	number of simulations per parameter set
dt	0.0001	τ	simulation time step
t_{final}	50	τ	total simulation time
dx	0.1	λ	spacing between lattice sites
N_{sites}	128	1	number of lattice sites
α	$[0, 1, \ldots, 25]$	τ^{-1}	driving frequency
σ_ω	1.57	τ^{-1}	width of Gaussian used to smooth in the temporal dimension
σ_k	1.47	λ^{-1}	width of Gaussian used to smooth in the spatial dimension

Supplementary Table 1. **Gaussian field simulation parameters.** Simulations of the Gaussian fields use an Euler-Maruyama algorithm to integrate the equations of motion. Time and space are scaled by $\tau = (Dr)^{-1}$ and $\lambda = r^{-1/2}$. The simulation is performed on a periodic, 1-dimensional lattice.

name	value	units	description
N_{sim}	50	1	number of simulations per parameter set
t_{final}	5000	τ	total time of simulation
k_1^+	1	τ^{-1}	forward reaction rate for reaction 1
k_1^-	0.5	τ^{-1}	reverse reaction rate for reaction 1
k_2^+	2	τ^{-1}	forward reaction rate for reaction 2
k_2^-	0.5	τ^{-1}	reverse reaction rate for reaction 2
k_3^+	2	τ^{-1}	forward reaction rate for reaction 3
k_3^-	0.5	τ^{-1}	reverse reaction rate for reaction 3
A	100	1	number of chemical species in reaction volume
V	100	1	reaction volume, used for calculated propensities in Gillespie algorithm

Supplementary Table 2. **Brusselator simulation parameters.** Simulations of the Brusselator are done using a Gillespie algorithm [1]. Time is non-dimensionalized by $\tau = \left(k_1^+\right)^{-1}$. The strength of external driving is given by $\Delta\mu = \log\left((bk_2^+k_3^+)(ck_2^-k_3^-)^{-1}\right)$, where b and c are the concentrations of B and C, respectively. Values of B and C are changed to give driving strengths $\Delta\mu \in [-2, 8]$ with step size 0.1, while keeping $\sqrt{(bk_2^+k_3^+)(ck_2^-k_3^-)} = 1$, with 1 an arbitrarily chosen constant. The EPR plot in Fig. 2c uses varying smoothing widths. When $\Delta\mu < 5$, $\sigma = 1.26$. When $\Delta\mu \in [5, 5.8]$, $\sigma = 0.063$. When $\Delta\mu > 5.8$, $\sigma = 0.031$. The EPF plot shown in Fig. 2b uses a smoothing width of $\sigma = 0.126$. Different system volumes are used in Fig. 3.

name	value	units	description
N_{sim}	10	1	number of simulations per parameter set
N_{c}	64	1	number of lattice sites
D_X	1	$\lambda^2\tau^{-1}$	diffusion constant of chemical species X
D_Y	0.1	$\lambda^2\tau^{-1}$	diffusion constant of chemical species Y
t_{final}	100	τ	total time of simulation
k_1^+	1	τ^{-1}	forward reaction rate for reaction 1
k_1^-	0.5	τ^{-1}	reverse reaction rate for reaction 1
k_2^+	2	τ^{-1}	forward reaction rate for reaction 2
k_2^-	0.5	τ^{-1}	reverse reaction rate for reaction 2
k_3^+	2	τ^{-1}	forward reaction rate for reaction 3
k_3^-	0.5	τ^{-1}	reverse reaction rate for reaction 3
A	100	1	number of chemical species in reaction volume
C	400	1	number of chemical species in reaction volume
V	100	1	reaction volume of each compartment, used to calculated propensities

Supplementary Table 3. **Reaction-diffusion Brusselator simulation parameters.** To add diffusion to the Brusselator, we employ a compartment-based Gillespie algorithm. Time is non-dimensionalized by $\tau = \left(k_1^+\right)^{-1}$, and space is non-dimensionalized by using the distance between each compartment, $\lambda = h = 1$.

$\Delta\mu$	$(\sigma_\omega, \sigma_q)$
$\in [-1, -0.5]$	(7, 1)
$\in [-0.5, 0.5]$	(14, 2)
$\in [0.5, 1]$	(7, 1)
$\in [1, 5.65]$	(5, 0.5)
$= 5.7$	(3.5, 0.4)
$= 5.8$	(1.75, 0.3)
$= 5.9$	(0.7, 0.1)
$= 6.0$	(0.35, 0.05)
$= 6.1$	(0.14, 0.02)
≥ 6.2	(0.035, 0.005)

Supplementary Table 4. **Smoothing widths as a function of driving force.** This table gives the smoothing widths $(\sigma_\omega, \sigma_q)$ as a function of chemical driving force $\Delta\mu$ used in Figure 4c in the main text. Figure 5 in the main text uses a smoothing width of $(\sigma_\omega, \sigma_q) = (0.07, 0.1)$ for all $\Delta\mu$.

Parameters			
Name	Value	units	Description
N_{sim}	64	1	number of simulations per parameter set
N_{dim}	2	1	number of dimensions of the simulation
dt	0.001	τ	simulation time step
t_{final}	100	τ	total simulation time
α	$[0, 1, \ldots, 10]$	τ^{-1}	strength of driving

Supplementary Table 5. **Driven Brownian particle simulation parameters** Simulations of the driven Brownian particle shown in the Supplementary Material use an Euler-Maruyama algorithm to integrate the equations of motion. Time is scaled by $\tau = \gamma/k$, which also scales the driving force. The variables have units of length and are scaled by $\lambda = \sqrt{D\gamma/k}$. Supplementary Figure 8 and Supplementary Figure 9 use a smoothing width of $\sigma = 1.88$

SUPPLEMENTARY NOTE 1: ENTROPY PRODUCTION FACTOR DERIVATION

Consider a system described by a set of real random scalar variables tracing some path through phase space, $\mathbf{X} = \{X^i(t)\}$ with Fourier transforms given by $x^i(\omega)$. Assuming the variables to be Gaussian distributed with real space covariance function with time translation invariance, $\langle X^i(t)X^j(t')\rangle = C^{ij}(t-t')$, the probability of observing a particular path is given in frequency space as

$$P[\mathbf{x}] = \frac{1}{Z}\exp\left[-\frac{1}{2}\int_{-\infty}^{\infty}\frac{d\omega}{2\pi}C^{-1}_{ij}(\omega)x^i(-\omega)x^j(\omega)\right] \tag{1}$$

where Z is the partition function and $C^{ij}(\omega) = \mathcal{F}\left[C^{ij}(t-t')\right]$ with $C^{-1}_{ij} \equiv (C^{-1})_{ij}$. The reverse path is given by Equation 1 with $\omega \to -\omega$ in the argument of C^{-1}_{ij}.

$$\widetilde{P}[\mathbf{x}] = \frac{1}{\widetilde{Z}}\exp\left[-\frac{1}{2}\int_{-\infty}^{\infty}\frac{d\omega}{2\pi}C^{-1}_{ij}(-\omega)x^i(-\omega)x^j(\omega)\right]. \tag{2}$$

To make the following calculations easier, we consider a discrete case for a finite time series of length $T = Ndt$ with sampling rate dt. In this case, Equation 1 is written as

$$P[\mathbf{x}] = \frac{1}{Z}\prod_{n=1}^{T/dt}\exp\left[-\frac{1}{2T}\left[C^{-1}(\omega_n)\right]_{ij}x^i(-\omega_n)x^j(\omega_n)\right] \tag{3}$$

where $\omega_n = 2\pi n/T$. Equation 2 is written similarly. We then have

$$\ln\left(\frac{P}{\widetilde{P}}\right) = \ln\left(\frac{\widetilde{Z}}{Z}\right) + \frac{1}{2T}\sum_{n=1}^{T/dt}\left[C^{-1}(-\omega_n) - C^{-1}(\omega_n)\right]_{ij}x^i(-\omega_n)x^j(\omega_n) \tag{4}$$

Using the fact that, for a finite signal of length T, $\langle x_i(\omega_n)x_j(-\omega_m)\rangle = T\delta_{nm}C_{ij}(\omega_n)$, the KL-divergence is then

$$D_{\mathrm{KL}}\left(P[\mathbf{x}]\,\middle\|\,\widetilde{P}[\mathbf{x}]\right) = \left\langle \ln\left(\frac{P}{\widetilde{P}}\right)\right\rangle_P \tag{5}$$

$$= \sum_{\mathbf{x}} P\ln\left(\frac{P}{\widetilde{P}}\right) \tag{6}$$

$$= \ln\left(\frac{\widetilde{Z}}{Z}\right) + \frac{1}{2}\sum_n\left[C^{-1}(-\omega_n) - C^{-1}(\omega_n)\right]_{ij}C^{ji}(\omega_n) \tag{7}$$

where $\sum_{\mathbf{x}}$ is a sum over all possible paths. The entropy production rate is then given by

$$\dot{S} = \lim_{T\to\infty}\frac{1}{2T}\sum_n\left[C^{-1}(-\omega_n) - C^{-1}(\omega_n)\right]_{ij}C^{ji}(\omega_n) = \lim_{T\to\infty}\frac{1}{T}\sum_n\mathcal{E}(\omega_n), \tag{8}$$

where we have introduced the entropy production factor, $\mathcal{E}$, and dropped the ratio of the partition functions as they will contribute 0 to the EPR when multiplied by $1/T$ and taking the $T \to \infty$ limit. In the limit taken, the sum becomes an integral, $\sum_n \to T/2\pi \int d\omega$, which brings us to our first main result

$$\dot{S} = \int_{-\infty}^{\infty} \frac{d\omega}{2\pi}\, \mathcal{E}(\omega) \tag{9}$$

$\mathcal{E}$ can also be rewritten as a $\mathcal{E}(\omega) = \mathrm{Tr}\{\mathbf{C}(\omega)\,[\mathbf{C}^{-1}(-\omega) - \mathbf{C}^{-1}(\omega)]\}/2$.

In the same spirit as above, we derive a similar expression for a set of real-valued random fields, $\boldsymbol{\eta}(\mathbf{r},t) = \{\phi^i(\mathbf{r},t) | \mathbf{r} \in \mathbb{R}^d\}$. The probability of following a path is given by

$$P[\boldsymbol{\eta}] = \frac{1}{Z} \exp\left[-\frac{1}{2} \iint \frac{d\omega}{2\pi} \frac{d^d\mathbf{q}}{(2\pi)^d} C_{ij}^{-1}(\mathbf{q},\omega) \phi^i(-\mathbf{q},-\omega) \phi^j(\mathbf{q},\omega)\right], \tag{10}$$

where the covariance function is defined as $\langle \phi^i(\mathbf{q},\omega)\phi^j(\mathbf{q}',\omega')\rangle = C^{ij}(\mathbf{q},\omega)\delta^d(\mathbf{q}+\mathbf{q}')\ \delta(\omega+\omega')$. Under time reversal, $\mathbf{q}$ is invariant but ω switches sign. Thus, $P[\widetilde{\boldsymbol{\eta}}]$ is given by

$$P[\widetilde{\boldsymbol{\eta}}] = \frac{1}{Z} \exp\left[-\frac{1}{2} \iint \frac{d\omega}{2\pi} \frac{d^d\mathbf{q}}{(2\pi)^d} C_{ij}^{-1}(\mathbf{q},-\omega) \phi^i(-\mathbf{q},-\omega) \phi^j(\mathbf{q},\omega)\right]. \tag{11}$$

We assume that the field is sampled in time with resolution dt for a time T (i.e. $\Delta\omega = 2\pi/T$) and each dimension of space is sampled with resolution dx_i for a length L_i (i.e. $\Delta k_i = 2\pi/L_i$), giving the discretized path probability functional

$$P[\boldsymbol{\eta}] = \frac{1}{Z} \prod_{n=0}^{T/dt} \prod_{m_1=0}^{L_1/dx_1} \cdots \prod_{m_d=0}^{L_d/dx_d} \exp\left[-\frac{1}{2TV}\ C_{ij}^{-1}(\mathbf{q}_m,\omega_n) \phi^i(-\mathbf{q}_m,-\omega_n) \phi^j(\mathbf{q}_m,\omega_n)\right], \tag{12}$$

where $\mathbf{q}_m \equiv (k_{1_{m_1}}, k_{2_{m_2}}, \ldots, k_{d_{m_d}})$ and V is the total volume. A similar expression exists for $P[\widetilde{\boldsymbol{\eta}}]$. From these expressions, we have (ignoring the partition functions that will add zero once we take the $T \to \infty$ limit)

$$\ln\left(\frac{P}{\widetilde{P}}\right) = \frac{1}{2VT} \sum_{n=0}^{T/dt} \sum_{m_1=0}^{L_1/dx_1} \cdots \sum_{m_d=0}^{L_d/dx_d} \left[C_{ij}^{-1}(\mathbf{q}_m,-\omega_n) - C_{ij}^{-1}(\mathbf{q}_m,\omega_n)\right] \phi^i(-\mathbf{q}_m,-\omega_n)\phi^j(\mathbf{q}_m,\omega_n). \tag{13}$$

Taking the average with respect to P and noting that $\langle \phi^i(\mathbf{q}_m,\omega_n)\phi^j(-\mathbf{q}_{m'},-\omega_{n'})\rangle = TV\delta_{mm'}\delta_{nn'}C^{ij}(\mathbf{q}_m,\omega_n)$ for finite signals, we have

$$D_{\mathrm{KL}}\left(P[\boldsymbol{\psi}] \parallel P[\widetilde{\boldsymbol{\eta}}]\right) = \frac{1}{2} \sum_{n=0}^{T/dt} \sum_{m_1=0}^{L_1/dx_1} \cdots \sum_{m_d=0}^{L_d/dx_d} \left[C^{-1}(\mathbf{q}_m,-\omega_n) - C^{-1}(\mathbf{q}_m,\omega_n)\right]_{ij} C^{ji}(\mathbf{q}_m,\omega_n). \tag{14}$$

This gives an entropy production rate of

$$\dot{S} = \lim_{T\to\infty} \frac{1}{2T} \sum_{n=0}^{T/dt} \sum_{m_1=0}^{L_1/dx_1} \cdots \sum_{m_d=0}^{L_d/dx_d} \mathcal{E}(\mathbf{q}_m,\omega_n), \tag{15}$$

again introducing the EPF for fields, $\mathcal{E}(\mathbf{q}, \omega)$. Passing to the continuum limit, we have

$$\dot{S} = V \int_{-\infty}^{\infty} \frac{d\omega}{2\pi} \frac{d^d q}{(2\pi)^d} \mathcal{E}(\mathbf{q}, \omega), \tag{16}$$

The EPR density is given by $\dot{s} = \dot{S}V^{-1}$. As with the case of random variables, $\mathcal{E}$ can be rewritten as a trace, $\mathcal{E}(\mathbf{q}, \omega) = \mathrm{Tr}\{[\mathbf{C}^{-1}(\mathbf{q}, -\omega) - C^{-1}(\mathbf{q}, \omega)]\, \mathbf{C}(\mathbf{q}, \omega)\}/2$.

SUPPLEMENTARY NOTE 2: GAUSSIAN APPROXIMATION LOWER BOUNDS $\dot{S}$

The KL divergence in Eq. 1 is an exact expression for the entropy production rate provided that the observed set of variables, $\{x^\mu\}$, contains every non-equilibrium degree of freedom present in the system. In practice, one only has access to a subset of those degrees of freedom, making the measured KL divergence a lower bound on the entropy production rate. Here, we show that the Gaussian assumption for $P[\mathbf{X}]$ provides another lower bound on the irreversibility measured on the scale of the observed mesoscale trajectories.

The proof relies on the data processing inequality [2], which states that any transformation of variables $F : x^\mu \to y^\mu$ will lower the relative entropy between two distributions over both sets of variables,

$$D_{\mathrm{KL}}\left(P\left[\{x^\mu\}\right] || Q\left[\{x^\mu\}\right]\right) \geq D_{\mathrm{KL}}\left(P\left[\{y^\mu\}\right] || Q\left[\{y^\mu\}\right]\right). \tag{17}$$

Intuitively, it states that any processing of an observation $\{x^\mu\}$ makes it more difficult to determine whether it came from P or Q. Our strategy will be to choose a transformation that will turn any distribution over x^μ into a Gaussian distribution over y^μ. In our case, our observables are the frequency space variables $x^\mu(\mathbf{q}, \omega)$, and the transformation is a multiplication of by a random phase field $\theta(\mathbf{q}, \omega)$, i.e. $x^\mu(\mathbf{q}, \omega) \to x^\mu(\mathbf{q}, \omega) e^{i\theta(\mathbf{q}, \omega)}$. This random phase, when integrated over frequency space, will make all correlations zero except for the two-point correlation function due to the fact that the variables in real space are real, making the two-point correlation equal to $\langle x^\mu (x^\mu)^* \rangle$, cancelling the random phase. Thus, the transformed variables are described by a Gaussian distribution (defined as the distribution whose only non-zero cumulants are the first and second), and the data processing inequality guarantees that this provides a lower bound to the KL divergence over the original distributions.

SUPPLEMENTARY NOTE 3: NUMERICAL CALCULATION OF $\mathcal{E}$ AND $\dot{S}$

While we were able to drop the partition functions in the analytic calculations above after taking the $T \to \infty$ limit and assuming translation and rotation invariance, the case of finite, discrete data

requires greater care due to the presence of noise. The partition function for Equation 10 is given by

$$Z = \exp\left(\frac{VT}{2}\iint \frac{d\omega}{2\pi}\frac{d\mathbf{q}}{2\pi}\ \ln\left[\det C(\mathbf{q},\omega)\right]\right). \tag{18}$$

and the EPR density is given by

$$\dot{s} = \frac{1}{2}\iint \frac{d^d\mathbf{q}}{(2\pi)^d}\frac{d\omega}{2\pi}\left[\ln\left(\frac{\det C(\mathbf{q},-\omega)}{\det C(\mathbf{q},\omega)}\right) + \left[C^{-1}(\mathbf{q},-\omega) - C^{-1}(\mathbf{q},\omega)\right]_{ij} C^{ji}(\mathbf{q},\omega)\right]. \tag{19}$$

In discrete form,

$$\dot{s} = \frac{1}{2TV}\sum_{m,n}\left[\ln\left(\frac{\det C(\mathbf{q}_m,-\omega_n)}{\det C(\mathbf{q}_m,\omega_n)}\right) + \left[C^{-1}(\mathbf{q}_m,-\omega_n) - C^{-1}(\mathbf{q}_m,\omega_n)\right]_{ij} C^{ji}(\mathbf{q}_m,\omega_n)\right] \tag{20}$$

For random variables, the equivalent of Equation 18 has an integral over only ω in the argument of the exponential resulting in the same value of $\tilde{Z}$ since the integral is over all ω. This gives $\log(\frac{\tilde{Z}}{Z}) = 0$, allowing one to drop the partition functions even for finite trajectories. However, for finite trajectories of fields, the partition functions are required to maintain the positivity of the relative entropy used to define $\dot{s}$.

SUPPLEMENTARY NOTE 4: DERIVATION OF BIAS IN $\dot{S}$ AND $\mathcal{E}$

We now turn to the problem of estimating the bias in our measured entropy production rate. For this, we assume that we have an equilibrium process and calculate what the average measured entropy production rate is, representing the systematic overestimation of our estimator. We work in coordinates where the covariance matrix is the identity, $C^{\mu\nu} = \delta^{\mu\nu}$. Due to a combination of measurement errors and only having a finite time series, we will measure a matrix that deviates from the identity by

$$\widetilde{C}^{\mu\nu} = \delta^{\mu\nu} + \widetilde{R}^{\mu\nu} + i\widetilde{A}^{\mu\nu}, \tag{21}$$

where $\widetilde{R}^{\mu\nu}(\omega)$ and $\widetilde{A}^{\mu\nu}(\omega)$ are elements of a symmetric and anti-symmetric $D \times D$ matrix, respectively, each assumed to be much smaller than 1. The anti-symmetric contribution must be purely imaginary because $\widetilde{C}^{\mu\nu}$ is a Hermitian matrix by definition. Further, we have $R^{\mu\nu}(-\omega) = R^{\mu\nu}(\omega)$ and $A^{\mu\nu}(-\omega) = -A^{\mu\nu}(\omega)$. For notational simplicity, we define $\widetilde{M}^{\mu\nu} \equiv \delta^{\mu\nu} + \widetilde{R}^{\mu\nu}$ and therefore $\hat{\mathbf{C}} = \hat{\mathbf{M}} + i\hat{\mathbf{A}}$.

To calculate the EPR, we need to calculate the EPF, $\mathcal{E} = \mathrm{Tr}\{\mathbf{C}(\omega)\left[\mathbf{C}^{-1}(-\omega) - \mathbf{C}^{-1}(\omega)\right]\}$. We approximate $\hat{\mathbf{C}}^{-1}$ as

$$\hat{\mathbf{C}}^{-1} = (\hat{\mathbf{M}} + i\hat{\mathbf{A}})^{-1} \approx \hat{\mathbf{M}}^{-1} - i\hat{\mathbf{M}}^{-1}\hat{\mathbf{A}}\hat{\mathbf{M}}^{-1}. \tag{22}$$

Then, $\mathbf{C}^{-1}(-\omega) - \mathbf{C}^{-1}(\omega) = 2i\hat{\mathbf{M}}^{-1}\hat{\mathbf{A}}\hat{\mathbf{M}}^{-1}$. Multiplying by $\hat{\mathbf{C}}$,

$$\mathbf{C}(\omega)\left[\mathbf{C}^{-1}(-\omega) - \mathbf{C}^{-1}(\omega)\right]\} = 2i\hat{\mathbf{M}}^{-1}\hat{\mathbf{A}}\hat{\mathbf{M}}^{-1}\hat{\mathbf{M}} - 2\hat{\mathbf{M}}^{-1}\hat{\mathbf{A}}\hat{\mathbf{M}}^{-1}\hat{\mathbf{A}} \tag{23}$$

Taking the trace of Equation 23, the first term is an asymmetric matrix with zero trace. By writing $\hat{\mathbf{M}} = \mathbb{I} + \hat{\mathbf{R}}$, we approximate $\hat{\mathbf{M}}^{-1} \approx \mathbb{I} - \hat{\mathbf{R}}$, and the second term is approximately as $\hat{\mathbf{A}}^2 + \mathcal{O}(\hat{\mathbf{A}}^2\hat{\mathbf{R}} + \hat{\mathbf{A}}\hat{\mathbf{R}}\hat{\mathbf{A}})$. Thus, to lowest order we have

$$\mathcal{E} = \mathrm{Tr}\{\mathbf{C}(\omega)\left[\mathbf{C}^{-1}(-\omega) - \mathbf{C}^{-1}(\omega)\right]\} \approx -\mathrm{Tr}\left(\hat{\mathbf{A}}^2\right) = 2\sum_{\mu>\nu}(\hat{A}^{\mu\nu})^2. \tag{24}$$

Assuming each element of $\mathbf{A}$ is an indepenent and identically distributed random variable, we can write the average EPF measured at equilibrium as

$$\langle\mathcal{E}\rangle_{\mathrm{eq}} = 2\frac{M(M-1)}{2}\left\langle(\hat{A}^{\mu\nu})^2\right\rangle = \frac{M(M-1)}{2}. \tag{25}$$

We calculated $\left\langle(\widetilde{A}^{\mu\nu})^2\right\rangle$ as follows:

$$\begin{aligned}
\left\langle(\widetilde{A}^{\mu\nu})^2\right\rangle &= \left\langle[\mathrm{Im}(x^\mu x^{\nu*})]^2\right\rangle && (26)\\
&= \left\langle[\mathrm{Re}(x^\mu)\mathrm{Im}(x^{\nu*}) + \mathrm{Im}(x^\mu)\mathrm{Re}(x^{\nu*})]^2\right\rangle && (27)\\
&= \left\langle[\mathrm{Re}(x^\mu)\mathrm{Im}(x^{\nu*})]^2 + [\mathrm{Im}(x^\mu)\mathrm{Re}(x^{\nu*})]^2 + \text{cross-terms}\right\rangle && (28)\\
&= \frac{1}{2} && (29)
\end{aligned}$$

The cross-terms average to zero because, in our choice of coordinate system, $|x^\mu|^2 = 1$, so the real and imaginary parts of x^μ are equally distributed along the unit circle. In addition, x^μ and x^ν should be uncorrelated (recall that we are working at equilibrium). The first and second term each have both a real and an imaginary part squared, each of which is always positive and on average equal to $\frac{1}{2}$. Thus, each term is $\frac{1}{4}$ and adds to $\frac{1}{2}$.

To estimate $\hat{\dot{S}}$, we smooth $\widetilde{\mathcal{E}}$ and integrate over all frequencies. We calculate $\left\langle(\hat{A}^{\mu\nu})^2\right\rangle$ as

$$(\hat{A}^{\mu\nu})^2 = \left(\sum_{\omega_i}\Delta\omega\frac{\exp\left[-\frac{(\omega_i-\omega_n)^2}{2\sigma^2}\right]}{\sqrt{2\pi\sigma^2}}\widetilde{A}^{\mu\nu}(\omega_i)\right)^2 \tag{30}$$

$$\begin{aligned}
\left\langle(\hat{A}^{\mu\nu})^2\right\rangle &= \sum_{\omega_i}(\Delta\omega)^2\frac{\exp\left[-\frac{(\omega_i-\omega_n)^2}{\sigma^2}\right]}{2\pi\sigma^2}\left\langle(\hat{A}^{\mu\nu})^2(\omega_i)\right\rangle && (31)\\
&\approx \frac{\Delta\omega}{4\pi\sigma^2}\int d\omega\,\exp[-\omega^2/\sigma^2] = \frac{\Delta\omega}{4\pi\sigma^2}\sqrt{\pi\sigma^2} && (32)\\
&= \frac{\sqrt{\pi}}{2T\sigma}. && (33)
\end{aligned}$$

We used $\left\langle \widetilde{A}^{\mu\nu}(\omega_i)\widetilde{A}^{\mu\nu}(\omega_j)\right\rangle = \left\langle (\widetilde{A}^{\mu\nu})^2(\omega_i)\right\rangle \delta_{ij}$ in the second line, passed to an integral using one of the integration measures $\Delta\omega$ in the third line, and substituted $\Delta\omega = 2\pi(T)^{-1}$ in the fourth line. Finally, we arrive at

$$\dot{S}_{\text{eq}} = 2\frac{M(M-1)}{2}\int_{-\omega^{\max}}^{\omega^{\max}} \frac{d\omega}{2\pi}\left\langle (\hat{A}^{\mu\nu})^2\right\rangle = \frac{M(M-1)}{2}\frac{\omega^{\max}}{T\sigma\sqrt{\pi}} \tag{34}$$

If we also average the covariance functions over N independent trajectories with the same dynamics, this bias is further reduced, leaving us with our final estimate of the bias in our entropy production rate estimator

$$\dot{S}_{\text{eq}} = \frac{1}{N}\frac{M(M-1)}{2}\frac{\omega^{\max}/\sigma}{T\sqrt{\pi}} \tag{35}$$

Following the same line of reasoning for a set of M fields in $d+1$ dimensions, a similar expression can be derived. We again write $C^{\mu\nu}(\mathbf{q},\omega) = \mathbb{I} + R^{\mu\nu}(\mathbf{q},\omega) + iA^{\mu\nu}(\mathbf{q},\omega)$. Extra care must be taken in the field case because Equation 16 does not have the same symmetries as Equation 9. Specifically, while $R^{\mu\nu}(-\mathbf{q},-\omega) = R^{\mu\nu}(\mathbf{q},\omega)$ and $A^{\mu\nu}(-\mathbf{q},-\omega) = -A^{\mu\nu}(-\mathbf{q},-\omega)$, nothing can be said *a priori* about $R(\mathbf{q},-\omega)$ or $A(\mathbf{q},-\omega)$.

In order to calculate the bias, we will calculate the mean of the spatiotemporal entropy production factor, $\mathcal{E} = \mathrm{Tr}\{[\mathbf{C}^{-1}(\mathbf{q},-\omega) - \mathbf{C}^{-1}(\mathbf{q},\omega)]\,\mathbf{C}(\mathbf{q},\omega)\}$. The calculation is tedious, so we only report the result here:

$$\langle\mathcal{E}\rangle_{\text{eq}} = \left\langle \mathrm{Tr}\left[\mathbf{R}^2 - \mathbf{A}^2\right]\right\rangle \tag{36}$$

$$= M(M-1)\left(\left\langle (R^{\mu\nu})^2\right\rangle + \left\langle (A^{\mu\nu})^2\right\rangle\right) + M\left\langle (R^{\mu\mu})^2\right\rangle \tag{37}$$

$$= M(M-1) + \frac{3M}{4} \tag{38}$$

We arrived at this by using the fact that $\mathrm{Tr}\left(\mathbf{A}^2\right) = \sum_{\mu\neq\nu}(A^{\mu\nu})^2$ and $\mathrm{Tr}\left(\mathbf{R}^2\right) = \sum_{\mu}(R^{\mu\mu})^2 + \sum_{\mu\neq\nu}(R^{\mu\nu})^2$ for an asymmetric and symmetric matrix, respectively, in addition to the assumption that every matrix element is an independent and identically distributed random variable. As before, $\langle (A^{\mu\nu})^2\rangle = 1/2 = \langle (R^{\mu\nu})^2\rangle$. Now turning to the diagonal elements of $\mathbf{R}$,

$$\left\langle (R^{\mu\mu})^2\right\rangle = \left\langle [1 - \mathrm{Re}\left(\phi^{\mu}\phi^{\mu *}\right)]^2\right\rangle \tag{39}$$

$$= 1 + \left\langle \mathrm{Re}\left(\phi^{\mu}\right)^2 \mathrm{Im}\left(\phi^{\mu}\right)^2\right\rangle + \left\langle \mathrm{Re}\left(\phi^{\mu}\right)^4\right\rangle + \left\langle \mathrm{Im}\left(\phi^{\mu}\right)^4\right\rangle - 2\left\langle \mathrm{Re}\left(\phi^{\mu}\right)^2 + \mathrm{Im}\left(\phi^{\mu}\right)^2\right\rangle \tag{40}$$

$$= \frac{3}{4}, \tag{41}$$

where we used $\langle x^4\rangle = 3\langle x^2\rangle^2$ for Gaussian variables and $\left\langle \mathrm{Re}\left(\phi^{\mu}\right)^2\right\rangle = \left\langle \mathrm{Im}\left(\phi^{\mu}\right)^2\right\rangle = 1/2$ in our choice of coordinate system.

Assuming the signals to have a total length in time of T and a total length in each spatial dimension of L_i, we smooth the spatiotemporal covariance function with a multivariate Gaussian of width σ_ω in the temporal dimension, and σ_{k_i} in each of the spatial dimensions, giving a factor of $\omega^{\max}\left(\sigma_\omega T\sqrt{\pi}\right)^{-1}$ for the temporal dimension and $q_i^{\max}\left(\sigma_{q_i} L_i\sqrt{\pi}\right)^{-1}$ for each spatial dimension. Putting all these results together, we have

$$\dot{s}_{\text{eq}} = \frac{1}{N}\left(\frac{M(M-1)}{2} + \frac{3M}{8}\right)\frac{\omega^{\max}}{T\sigma_\omega\sqrt{\pi}}\prod_{i=1}^{d}\frac{q_i^{\max}}{L_i\sigma_{q_i}\sqrt{\pi}} \tag{42}$$

SUPPLEMENTARY NOTE 5: ANALYTIC $\dot{S}$ OF COUPLED GAUSSIAN FIELDS

Consider the coupled equations of motion for the scalar fields ϕ and ψ in $d+1$ dimensions.

$$\partial_t\phi(\mathbf{x},t) = -D(r-\nabla^2)\phi - \alpha\psi + \sqrt{2D}\xi_\phi \tag{43}$$

$$\partial_t\psi(\mathbf{x},t) = -D(r-\nabla^2)\psi + \alpha\phi + \sqrt{2D}\xi_\psi. \tag{44}$$

with $\langle\xi^i(\mathbf{x},t)\xi^j(\mathbf{x}',t')\rangle = \delta^{ij}\delta(t-t')\delta^d(\mathbf{x}-\mathbf{x}')$. This is a Gaussian model with free energy

$$F = \int d^dx\,\left[\frac{r}{2}\left(\phi^2+\psi^2\right) + \frac{1}{2}\left(|\nabla\phi|^2 + |\nabla\psi|^2\right)\right]. \tag{45}$$

The interaction term cannot be written as a gradient of an energy, so we have

$$\partial_t\phi(\mathbf{x},t) = -D\frac{\delta F}{\delta\phi} - \alpha\psi + \sqrt{2D}\xi_\phi \tag{46}$$

$$\partial_t\psi(\mathbf{x},t) = -D\frac{\delta F}{\delta\psi} + \alpha\phi + \sqrt{2D}\xi_\psi. \tag{47}$$

Combining the two fields into a single vector, $\boldsymbol{\eta}(\mathbf{x},t) = (\phi(\mathbf{x},t),\psi(\mathbf{x},t))^T$, we write:

$$\partial_t\boldsymbol{\eta} = B\boldsymbol{\eta} + \sqrt{2D}\boldsymbol{\xi};\qquad B(\mathbf{x}) = \begin{pmatrix} -D(r-\nabla^2) & -\alpha \\ \alpha & -D(r-\nabla^2)\end{pmatrix}. \tag{48}$$

To get the cross-spectral density, we rewrite Equation 48 as an Ito stochastic differential equation:

$$d\boldsymbol{\eta} = B\boldsymbol{\eta}\,dt + \Xi\,d\mathbf{W}, \tag{49}$$

where $\mathbf{W}(\mathbf{x},t)$ is a multidimensional Wiener process in space and time with strength $\Xi^{ij} = \sqrt{2D}\delta^{ij}$. The eigenvalues of A have negative real parts, so a stationary solution exists. The cross-spectral density is [3]

$$C(\mathbf{q},\omega) = \left(B(\mathbf{q}) - i\omega\mathbb{I}\right)^{-1}\Xi\Xi^T\left(B(\mathbf{q}) + i\omega\mathbb{I}\right)^{-T}. \tag{50}$$

Noting $\mathbf{\Xi}\ \mathbf{\Xi}^T = 2D\mathbb{I}$, we have

$$C(\omega) = \frac{2D}{|\left(D(r+q^2)+i\omega\right)^2+\alpha^2)|^2}\begin{pmatrix}\left[D(r+q^2)\right]^2+\alpha^2+\omega^2 & i2\alpha\omega \\ -i2\alpha\omega & \left[D(r+q^2)\right]^2+\alpha^2+\omega^2\end{pmatrix}. \tag{51}$$

The inverse is given by

$$C^{-1}(\omega) = \frac{1}{2D}\begin{pmatrix}\left[D(r+q^2)\right]^2+\alpha^2+\omega^2 & -i2\alpha\omega \\ i2\alpha\omega & \left[D(r+q^2)\right]^2+\alpha^2+\omega^2\end{pmatrix}. \tag{52}$$

Finally, using Equation 16, we have

$$\dot{S} = V\iint_{-\infty}^{\infty}\frac{d\omega}{2\pi}\frac{d\mathbf{q}}{2\pi}\frac{8\alpha^2\omega^2}{|(D(r+q^2)+i\omega)^2+\alpha^2)|^2} = V\frac{\alpha^2}{D\sqrt{r}}. \tag{53}$$

Rearranging the denominator of the integrand of above gives $\mathcal{E}^{DGF}$ given in the main text.

We can alternatively calculate the entropy production rate by using the Onsager-Machlup functional [4] for the path probability functional $P[\boldsymbol{\eta}]$ in

$$\dot{S} = \lim_{T\to\infty}\frac{1}{T}\left\langle \ln\frac{P[\boldsymbol{\eta}]}{\widetilde{P}[\boldsymbol{\eta}]}\right\rangle \tag{54}$$

Writing it as a path $P[\boldsymbol{\eta}] \propto \exp(-\mathcal{A})$, where $\mathcal{A}$ is the action, this becomes

$$\dot{S} = \lim_{T\to\infty}\frac{1}{T}\left\langle \widetilde{\mathcal{A}} - \mathcal{A}\right\rangle, \tag{55}$$

where $\widetilde{\mathcal{A}}$ is the action under time-reversal. To calculate $\mathcal{A}$, we use standard path integral techniques, i.e. the Martin-Siggia-Rose formalism [5]. The idea is to try and find the expectation of some observable, O, over noise realizations.

$$\langle O[\boldsymbol{\eta}]\rangle_\xi = \int \mathcal{D}[\boldsymbol{\xi}]\ O[\boldsymbol{\eta}]P[\boldsymbol{\xi}]. \tag{56}$$

Since the noise is Gaussian, we have

$$P[\boldsymbol{\xi}] \propto \exp\left(\frac{1}{4D}\int d^dx\ dt\ \boldsymbol{\xi}^2\right) \tag{57}$$

(we use Einstein notation throughout). We then insert the most complicated expression for 1 ever written. Using the integral representation of the functional delta function, $\delta[f(x)] = \int \mathcal{D}[i\widetilde{f}]\exp[-\int dx\widetilde{f}(x)f(x)]$, we write

$$1 = \int\prod_j \mathcal{D}[\eta^j]\delta(\partial_t\eta^j - B^j_k\eta^k - \xi^j) \tag{58}$$

$$= \int\prod_j \mathcal{D}[\eta^j]\mathcal{D}[i\widetilde{\eta}_j]\exp - \int d^dx\ dt\left[\widetilde{\eta}_j\left(\partial_t\eta^j - B^j_k\eta^k - \xi^j\right)\right] \tag{59}$$

to get

$$\langle O[\boldsymbol{\eta}]\rangle_\xi = \int \mathcal{D}[\boldsymbol{\xi}] \prod_j \mathcal{D}[\eta^j]\mathcal{D}[i\widetilde{\eta}_j]\; O[\boldsymbol{\eta}] \exp\left[\frac{1}{4}\int d^dx\; dt \left(\xi^i \Xi^{-1}_{ij}\xi^j - 4\widetilde{\eta}_j\xi^j\right) - \widetilde{\eta}_j\left(\partial_t\eta^j - B^j_k\eta^k\right)\right]. \tag{60}$$

Completing the square in ξ and doing the Gaussian integrals, we get

$$\langle O[\boldsymbol{\eta}]\rangle_\xi = \int \prod_j \mathcal{D}[\eta^j]\mathcal{D}[i\widetilde{\eta}_j]\; O[\boldsymbol{\eta}] \times \exp\left\{-\int d^dx\; dt \left[\widetilde{\eta}_j\left(\partial_t\eta^j - B^j_k\eta^k\right) - \widetilde{\eta}_j \Xi^{jk}\widetilde{\eta}_k\right]\right\}. \tag{61}$$

Doing the integrals over the response fields $\widetilde{\eta}$, we are left with

$$\langle O[\boldsymbol{\eta}]\rangle_\xi = \int \prod_j \mathcal{D}[\eta^j]\; O[\boldsymbol{\eta}]\; \exp(-\mathcal{A}[\boldsymbol{\eta}]) \tag{62}$$

where $\mathcal{A}$ is the Onsager-Machlup functional

$$\mathcal{A} = -\frac{1}{4D}\int d^dx\; dt\; \left(\partial_t\eta^j - B^j_k\eta^k\right)^2 \tag{63}$$

Noting that the only time asymmetric part of the action is $\partial_t\eta$, we can write

$$\mathcal{A} = -\frac{1}{4D}\int d^dx\; dt\; \left(\partial_t\phi + D\frac{\delta F}{\delta\phi} + \alpha\psi\right)^2 + \left(\partial_t\psi + D\frac{\delta F}{\delta\psi} - \alpha\phi\right)^2 \tag{64}$$

$$\widetilde{\mathcal{A}} = -\frac{1}{4D}\int d^dx\; dt\; \left(\partial_t\phi - D\frac{\delta F}{\delta\phi} - \alpha\psi\right)^2 + \left(\partial_t\psi - D\frac{\delta F}{\delta\psi} + \alpha\phi\right)^2 \tag{65}$$

Taking the difference $\widetilde{\mathcal{A}} - \mathcal{A}$, and noting that $(a+b)^2 - (a-b)^2 = 4ab$, we have

$$\widetilde{\mathcal{A}} - \mathcal{A} = -\frac{1}{D}\int d^dx dt\; \partial_t\phi\left(-D\frac{\delta F}{\delta\phi} - \alpha\psi\right) + \partial_t\psi\left(-D\frac{\delta F}{\delta\psi} + \alpha\phi\right) \tag{66}$$

In the Stratonovich convention, $dF/dt = \partial_t\phi\;(\delta F/\delta\phi) + \partial_t\psi\;(\delta F/\delta\psi)$, which will turn into a constant difference in free energies upon taking the time integral. This constant value will tend to zero as the limit $T \to \infty$ is taken. Further, there is a time-symmetric portion of the action that is being omitted due to the Jacobian factor in switching from an integral in $\boldsymbol{\xi}$ to $\boldsymbol{\eta}$ that also arises due to the Stratonovich discretization used throughout this article.

We find the entropy production rate to be

$$\dot{S} = \lim_{T\to\infty} \frac{\alpha}{DT}\int d^dx dt\; \left(\psi\dot{\phi} - \dot{\psi}\phi\right) \tag{67}$$

Plugging in the equations of motion, we find

$$\left\langle \dot{\psi}\phi \right\rangle = \left\langle -D\frac{\delta F}{\delta\psi}\phi - \alpha\phi^2 + \xi_\psi\phi \right\rangle = \left\langle -D\left[(r-\nabla^2)\psi\right]\phi - \alpha\phi^2 + \xi_\psi\phi\right\rangle \tag{68}$$

$$\left\langle \psi\dot{\phi} \right\rangle = \left\langle -D\frac{\delta F}{\delta\phi}\psi + \alpha\psi^2 + \xi_\phi\psi \right\rangle = \left\langle -D\left[(r-\nabla^2)\phi\right]\psi + \alpha\psi^2 + \xi_\phi\psi\right\rangle \tag{69}$$

Some care must be taken in evaluating the terms linear in the noise. If the Ito convention had been used, they would be trivially zero, but that is not the general case in the Stratonovich convention. However, as each field is multiplied by the opposite component of the noise, one can show that they indeed identically equal 0. Putting everything together, we have

$$-Dr\psi\phi + D(\nabla^2\psi)\phi + \alpha\phi^2 + Dr\phi\psi - D(\nabla^2\phi)\psi + \alpha\psi^2 \tag{70}$$

The two Laplacian terms will cancel under one integration by parts each, leaving us with

$$\dot{S} = \frac{\alpha^2}{D}\int d^dx \ \left\langle \phi^2 + \psi^2 \right\rangle \tag{71}$$

where we have replaced the time average with an ensemble average, assuming ergodicity. Assuming the system to be in the steady state, we integrate over the equal-time (i.e. $\omega = 0$) power spectrum of ϕ and ψ

$$\langle \phi(k)\phi(-k)\rangle = \int \frac{dk}{2\pi}\frac{1}{k^2+r} = \frac{1}{2\sqrt{r}}. \tag{72}$$

Using this expression for both ϕ and ψ in the equation for $\dot{S}$ above, we have

$$\dot{S} = \frac{\alpha^2}{D\sqrt{r}}V, \tag{73}$$

where V is the total volume of the space, and $\dot{s} = \dot{S}/V$.

SUPPLEMENTARY NOTE 6: $\mathcal{E}$ AND HARADA-SASA RELATION

Here, we show that $\mathcal{E}^{\mathrm{DGF}}$, written in Equation 7 of the main text, is equivalent to the spectral decomposition based on a generalized Harada-Sasa relation (GHSR) given in [6]. The non-equilibrium driving in Equation 48 comes from a rotational current between the two fields, rather than from a current derived from the gradient of a non-conservative chemical potential. This requires us to derive a slightly altered version of the GHSR than given in [6], largely following the steps outlined therein. In addition, our 2-field problem requires that the response and correlation functions be tensors, and the energy dissipation is related to the trace of their difference [7].

We consider the response of the fields $\boldsymbol{\eta} = (\phi, \psi)$ to a constant external field, $(h_\phi(\mathbf{r}), h_\psi(\mathbf{r}))$, changing the action to

$$\mathcal{A}^h = -\frac{1}{4D}\int d^dx\ dt\ \left(\partial_t\phi + D\frac{\delta F}{\delta\phi} + Dh_\phi + \alpha\psi\right)^2 + \left(\partial_t\psi + D\frac{\delta F}{\delta\psi} + Dh_\psi - \alpha\phi\right)^2 \tag{74}$$

The response function is upgraded to a response tensor, $\mathcal{R}$, with elements

$$R_{ij}(\mathbf{r}_1, \mathbf{r}_2, t) = -\left\langle \eta_i(\mathbf{r}_1, t)\frac{\delta\mathcal{A}^h}{\delta h_j(\mathbf{r}_2, 0)}\bigg|_{h_j=0}\right\rangle \tag{75}$$

To first order in each external field, the perturbation to the action is

$$\delta A^h = -\frac{1}{2}\int d^dxdt\ \left[h_\phi\left(\partial_t\phi + D\frac{\delta F}{\delta\phi} + \alpha\psi\right) + h_\psi\left(\partial_t\psi + D\frac{\delta F}{\delta\psi} - \alpha\phi\right)\right] \tag{76}$$

The trace of $\mathcal{R}$, taken at the same spatial location, is

$$\begin{aligned}\mathrm{Tr}\left(\mathcal{R}(\mathbf{r},\mathbf{r},t)\right) = -\frac{1}{2}\int d^dxdt\ \Big[\phi(\mathbf{r},t)\left(\partial_t\phi + D\frac{\delta F}{\delta\phi} + \alpha\psi\right)\Big|_{(\mathbf{r},0)} \\ +\psi(\mathbf{r},t)\left(\partial_t\psi + D\frac{\delta F}{\delta\psi} - \alpha\phi\right)\Big|_{(\mathbf{r},0)}\Big].\end{aligned} \tag{77}$$

The time-asymmetric part of the above equation can be written as

$$\begin{aligned}\mathrm{Tr}\left[\mathcal{R}(\mathbf{r},\mathbf{r},t) - \mathcal{R}(\mathbf{r},\mathbf{r},-t)\right] = &- \mathrm{Tr}\left[\partial_t C(\mathbf{r},\mathbf{r},t)\right] \\ &+\frac{\alpha}{2}\left\langle\left(\phi(\mathbf{r},t) - \phi(\mathbf{r},-t)\right)\psi(\mathbf{r},0) - \left(\psi(\mathbf{r},t) - \psi(\mathbf{r},-t)\right)\phi(\mathbf{r},0)\right\rangle,\end{aligned} \tag{78}$$

where the correlation matrix has elements $C_{ij}(\mathbf{r}_1,\mathbf{r}_2,t_1-t_2) = \langle\eta_i(\mathbf{r}_1,t_1)\eta_j(\mathbf{r}_2,t_2)\rangle$, equivalent to what was used above to define $\mathcal{E}$. We have also dropped the terms proportional to $\delta F/\delta\phi$ as they are constants that will be eliminated when we take the time derivative in the next line. Looking at Equation 67, we can rewrite the above as

$$\dot{S} = \frac{1}{D}\lim_{t\to 0}\int d^dxdt\ \partial_t\mathrm{Tr}\left[\mathcal{R}(\mathbf{r},\mathbf{r},t) - \mathcal{R}(\mathbf{r},\mathbf{r},-t) + \partial_t C(\mathbf{r},\mathbf{r},t)\right]. \tag{79}$$

Taking the Fourier transform, and denoting the imaginary part of $\mathcal{R}$ as $\widetilde{\mathcal{R}}$, we arrive at our modified GHSR

$$\dot{S} = \int\frac{d\omega}{2\pi}\frac{d\mathbf{q}}{(2\pi)^d}\sigma(\mathbf{q},\omega);\qquad \sigma(\mathbf{q},\omega) = \frac{\omega}{D}\mathrm{Tr}\left[\omega C(\mathbf{q},\omega) - 2\widetilde{\mathcal{R}}(\mathbf{q},\omega)\right]. \tag{80}$$

It now remains to check if $\sigma = \mathcal{E}$ for the driven Gaussian fields. As we have already calculated the correlation matrix, we are left to calculate $R_{ij} = \delta\langle\eta_i\rangle/\delta h_j$. Solving the perturbed versions of Equation 43 in frequency space and taking the mean, we find

$$\langle\phi\rangle = \frac{Dh_\phi + \alpha\langle\psi\rangle}{D(r+q^2) - i\omega} \tag{81}$$

$$\langle\psi\rangle = \frac{Dh_\psi - \alpha\langle\phi\rangle}{D(r+q^2) - i\omega}. \tag{82}$$

Plugging one solution into the other, we find the auto-responses to be equal to each other, giving

$$\mathrm{Tr}\left[\mathcal{R}\right] = \frac{2D\left(D(r+q^2) - i\omega\right)}{\left(D(r+q^2) - i\omega\right)^2 + \alpha^2} \to \mathrm{Tr}\left[\widetilde{\mathcal{R}}\right] = \frac{2D\omega\left(D^2(r+q^2)^2 - \alpha^2 + \omega^2\right)}{(\omega^2-\omega_0^2)^2 + (2r\omega)^2}, \tag{83}$$

where ω_0 is defined as in the main text, $\omega_0^2 = D^2(r+q^2)^2 + \alpha^2$. As can be seen in the previous section, the trace of the correlation function is

$$\mathrm{Tr}\left[C\right] = \frac{4D\left(D^2(r+q^2)^2 + \alpha^2 + \omega^2\right)}{(\omega^2-\omega_0^2)^2 + (2r\omega)^2}. \tag{84}$$

This finally gets us to our desired result

$$\sigma(\mathbf{q},\omega) = \frac{8\alpha^2\omega^2}{(\omega^2-\omega_0^2)^2+(2r\omega)^2} = \mathcal{E}^{\mathrm{DGF}}. \tag{85}$$

SUPPLEMENTARY NOTE 7: MACROSCOPIC BRUSSELATOR DYNAMICS

The reversible Brusselator model we consider in this paper is defined by

$$A \underset{k_1^-}{\overset{k_1^+}{\rightleftharpoons}} X; \quad B+X \underset{k_2^-}{\overset{k_2^+}{\rightleftharpoons}} Y+C; \quad 2X+Y \underset{k_3^-}{\overset{k_3^+}{\rightleftharpoons}} 3X, \tag{86}$$

where A, B, C are fixed external chemicals and the system is assumed to occur in a well-mixed vessel of volume V. Using mass action kinetics and writing lower-case letters as concentrations (e.g. $x \equiv X/V$), the macroscopic dynamics of the Brusselator are given by the coupled ODEs

$$\begin{aligned} \dot{x} &= k_1^+ a - k_1^- x - k_2^+ bx + k_2^- cy + k_3^+ x^2 y - k_3^- x^3 \\ \dot{y} &= k_2^+ bx - k_2^- cy - k_3^+ x^2 y + k_3^- x^3 \end{aligned} \tag{87}$$

Detailed balance holds when each reaction rate in Equation 86 is balanced, leading to the following equilibrium concentrations

$$X_{eq} = A\frac{k_1^+}{k_1^-} \tag{88}$$

$$Y_{eq} = X_{eq}\frac{Bk_2^+}{Ck_2^-} = X_{eq}\frac{k_3^-}{k_3^+}, \tag{89}$$

where the first and second equation for y_{eq} come from the k_2 and k_3 reactions, respectively. Using the two equations for y_{eq} gives us the condition for detailed balance given in the main text, $Bk_2^+k_3^+ = Ck_2^-k_3^-$.

The steady state values of (x, y) are given by setting the deterministic equations to 0, giving

$$x_{ss} = a\frac{k_1^+}{k_1^-} \tag{90}$$

$$y_{ss} = \frac{k_2^+ bx_{ss} + k_3^- x_{ss}^3}{k_2^- c + k_3^+ x_{ss}^2} \tag{91}$$

The relaxation matrix, R, that defines the stability of the steady state is given by expanding the deterministic equations to first order around their steady state values

$$R = \begin{pmatrix} \partial_x\dot{x} & \partial_y\dot{x} \\ \partial_x\dot{y} & \partial_y\dot{y} \end{pmatrix}\Bigg|_{ss} = \begin{pmatrix} -(k_1^- + bk_2^+) + 2k_3^+ x_{ss}y_{ss} - 3k_3^- x_{ss}^2 & k_2^- c + k_3^+ x_{ss}^2 \\ bk_2^+ - 2k_3^+ x_{ss}y_{ss} + 3k_3^- x_{ss}^2 & -(k_2^- c + k_3^+ x_{ss}^2) \end{pmatrix}. \tag{92}$$

The eigenvalues of R are given by solving its characteristic equation, giving

$$\lambda_\pm = \frac{\mathrm{Tr}(R)}{2} \pm \left[\left(\frac{\mathrm{Tr}(R)}{2}\right)^2 - \det(R)\right]^{1/2} \tag{93}$$

SUPPLEMENTARY NOTE 8: ALTERNATIVE $\dot{S}$ ESTIMATORS

One of the estimators is based on the thermodynamic uncertainty relation (TUR) [8, 9], which we implemented following [10]. To summarize the method, empirical phase space fluxes, $\mathbf{j}(\mathbf{x}, t)$ are integrated over space with a weighting vector field $\mathbf{d}(\mathbf{x})$ to give a macroscopic current, $j_{\mathbf{d}}$, that accumulates over an observation time:

$$j_{\mathbf{d}}(\tau_{\text{obs}}) = \int_0^{\tau_{\text{obs}}} dt \int d\mathbf{x}\, \mathbf{j}(\mathbf{x}, t) \cdot \mathbf{d}(\mathbf{x}). \tag{94}$$

The mean and variance of $j_{\mathbf{d}}$ are then used to estimate $\dot{S}$ as

$$\dot{S} \geq \dot{S}_{\text{TUR}} = \frac{2\langle j_{\mathbf{d}}\rangle}{\tau_{\text{obs}} \text{Var}(j_{\mathbf{d}})}. \tag{95}$$

The choice of weighting field $\mathbf{d}$ was shown in [10] to significantly affect the tightness of the bound given above. While they devised a Monte-Carlo procedure to minimize the mean-square error of $\dot{S}_{\text{TUR}}$, we opted to use a choice of $\mathbf{d}$ informed by the knowledge of the underlying dynamics. Namely, we chose $\mathbf{d} = (\dot{x}, \dot{y})$, defined in Equation 87.

The other estimator is based on the mean first passage time (MFPT) of an observable, $\mathcal{O}$, based on the method presented in [11]. The method involves measuring the average time, $\langle \tau_{\mathcal{O}} \rangle$, it takes for $\mathcal{O}$ to reach a threshold value $L(\alpha) = \ln((1-\alpha)/\alpha)$, where α is the fraction of false-positives (and false-negatives) deemed tolerable by the user. $\dot{S}$ is then bounded by:

$$\dot{S} \geq \frac{L(\alpha)(1-2\alpha)}{\langle \tau_{\mathcal{O}} \rangle}. \tag{96}$$

If $\mathcal{O}$ is chosen to be the log-likelihood ratio between the probabilities of observing a given time series conditioned on the hypothesis that the time series is played forwards or backwards, $\langle \tau_{\mathcal{O}} \rangle$ is minimized and the above bound is saturated. In order to restrict ourselves to dynamics observable in the (X, Y) plane, we choose $\mathcal{O}$ based on the winding number of the trajectory around the trajectory's center of mass, $2\pi w(t) = \theta(t)$, where $\theta(t) = \arctan\left(\frac{Y - \langle Y \rangle}{X - \langle X \rangle}\right)$, and $\theta(t)$ is measured cumulatively (e.g. two full counter-clockwise rotations give $\theta = 4\pi$). We then make the assumption that w is a drift-diffusion process that obeys the Langevin equation $\dot{w} = v + \sqrt{2D}\xi$, where ξ is unit variance Gaussian noise. The optimal observable for measuring the MFPT in this case is given by $\mathcal{O}(t) = \frac{v}{D}(w(t) - w(0))$, where $\langle w(T) \rangle = vT$ and $\text{Var}(w(T)) = 2DT$, T is the total observation time, and the mean and variance are calculated over many realizations of the same dynamics.

SUPPLEMENTARY NOTE 9: DRIVEN BROWNIAN PARTICLE

Here, we consider the particle version of the dynamics given by the Gaussian fields above. We consider an overdamped Brownian particle in a 2-dimensional harmonic trap with stiffness r subject to a non-conservative, rotational force. This is the particle version of the driven Gaussian fields considered above. The dynamics obey the Langevin equation

$$\dot{\mathbf{x}} = A\mathbf{x} + \sqrt{2}\boldsymbol{\xi}; \quad A = \begin{pmatrix} -r & -\alpha \\ \alpha & -r \end{pmatrix}, \tag{97}$$

where $\boldsymbol{\xi}$ is Gaussian white noise, $\langle \xi_i(t)\xi_j(t')\rangle = \delta_{ij}\delta(t-t')$. These linear dynamics exactly satisfy Equation 1 [3]. Using Equation 9 with the analytically calculated covariance functions, we find the exact EPF, intregrating to yield the EPR, in agreement with results from stochastic thermodynamics [12]

$$\mathcal{E}^{\mathrm{DBP}} = \frac{8\alpha^2\omega^2}{(\omega^2-\omega_0^2)^2 + (2r\omega)^2}, \quad \dot{S}^{\mathrm{DBP}}_{\mathrm{thry}} = \frac{2\alpha^2}{r} \tag{98}$$

The covariance functions are calculated in the same way we calculated the covariance functions for the driven Gaussian fields, simply replacing $D(r+q^2) \to r$. For the solution using stochastic thermodynamics, we write the Fokker-Planck equation associated with Equation 97 and solving for the steady state probability density, p_{ss} as well as the steady state current, j_{ss}, given by

$$p_{\mathrm{ss}}(\mathbf{x}) = \frac{r}{2\pi}e^{-rx^2/2}; \qquad j_{\mathrm{ss}}(\mathbf{x}) = \alpha\mathbf{x}p_{\mathrm{ss}}. \tag{99}$$

We can then calculate $\dot{S}$ as [12]

$$\dot{S} = \int d\mathbf{x}\, \frac{j_{\mathrm{ss}}^2}{p_{\mathrm{ss}}} = \frac{2\alpha^2}{r} \tag{100}$$

Similarly to $\mathcal{E}^{\mathrm{DGF}}$, $\mathcal{E}^{\mathrm{DBP}}$ is peaked at $\omega_0 = (r^2+\alpha^2)^{1/2}$ and decays as ω^2 for large ω. While multiple combinations of α and r can give the same value for $\dot{S}$, $\mathcal{E}$ distinguishes between equally dissipative trajectories in the shape and location of its peaks, giving information about the form of the underlying dynamics while retaining the same total EPR.

We see an excellent agreement between the measured $\hat{\mathcal{E}}$ and Equation 98, while also maintaining agreement with $\dot{S}^{\mathrm{DBP}}$ upon numerical integration of $\hat{\mathcal{E}}$ (Supplementary Figure 8). This remains true for 3, and 4 dimensional simulations (Supplementary Figure 9), highlighting our ability to estimate $\mathcal{E}$ for high dimensional data.

SUPPLEMENTARY REFERENCES

[1] Gillespie, D. T. Exact stochastic simulation of coupled chemical reactions. *J. Phys. Chem.* **81**, 2340–2361 (1977).

[2] Cover, T. M. & Thomas, J. A. *Elements of information theory* (Wiley-Interscience, 2006).
[3] Gardiner, C. W. *Stochastic Methods: A Handbook for the Natural and Social Sciences* (Springer Berlin, 2010), 4th edn.
[4] Onsager, L. & Machlup, S. Fluctuations and Irreversible Processes. *Phys. Rev.* **91**, 1505–1512 (1953).
[5] Martin, P. C., Siggia, E. D. & Rose, H. A. Statistical Dynamics of Classical Systems. *Phys. Rev. A* **8**, 423–437 (1973).
[6] Nardini, C. *et al.* Entropy production in field theories without time-reversal symmetry: Quantifying the non-equilibrium character of active matter. *Phys. Rev. X* **7** (2017).
[7] Harada, T. & Sasa, S.-i. Energy dissipation and violation of the fluctuation-response relation in nonequilibrium Langevin systems. *Phys. Rev. E* **73**, 026131 (2006).
[8] Barato, A. C. & Seifert, U. Thermodynamic Uncertainty Relation for Biomolecular Processes. *Phys. Rev. Lett.* **114**, 158101 (2015).
[9] Horowitz, J. M. & Gingrich, T. R. Proof of the finite-time thermodynamic uncertainty relation for steady-state currents. *Phys. Rev. E* **96**, 020103 (2017).
[10] Li, J., Horowitz, J. M., Gingrich, T. R. & Fakhri, N. Quantifying dissipation using fluctuating currents. *Nat. Comms.* **10**, 1666 (2019).
[11] Roldán, É., Neri, I., Dörpinghaus, M., Meyr, H. & Jülicher, F. Decision Making in the Arrow of Time. *Phys. Rev. Lett.* **115**, 250602 (2015). 1508.02018.
[12] Seifert, U. Stochastic thermodynamics, fluctuation theorems and molecular machines. *Rep. Prog. Phys.* **75**, 126001 (2012).
